# Supplementary material for: Immunoinformatics-Aided Analysis of RSV Fusion and Attachment Glycoproteins to Design a Potent Multi-Epitope Vaccine
Source: Vaccines (Basel). 2022 Aug 24;10(9):1381. doi: 10.3390/vaccines10091381 (PMC9502547; doi:10.3390/vaccines10091381)
Supplement: Supplementary file 1 [file vaccines-10-01381-s001.zip › vaccines-1824794-supplementary.pdf]

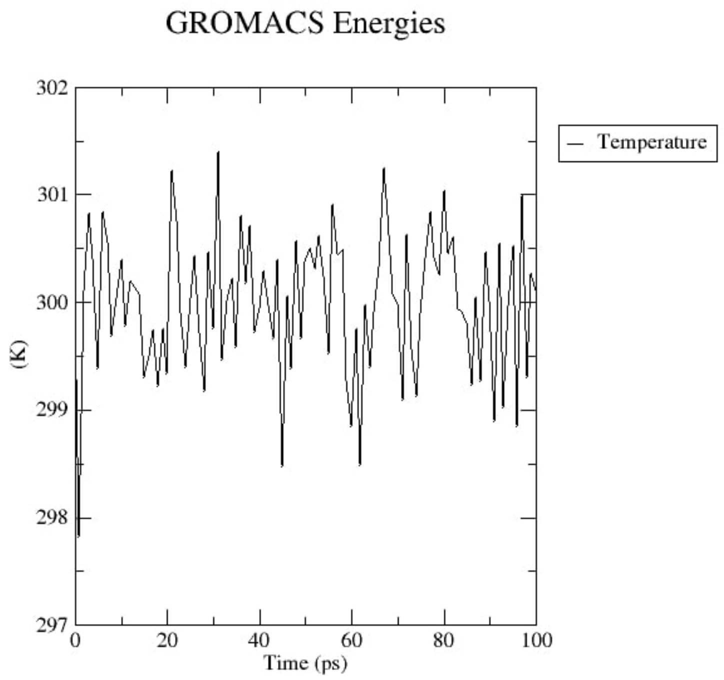

Figure S1. Graph showing minute fluctuations in the system's temperature during NVT equilibration steps.

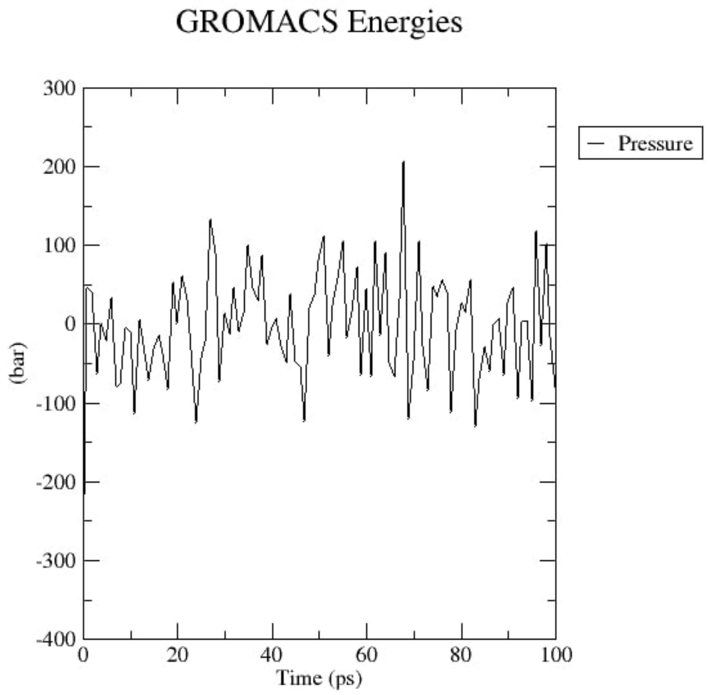

Figure S2. Graph showing minute fluctuations in the system's pressure during NPT equilibration steps.

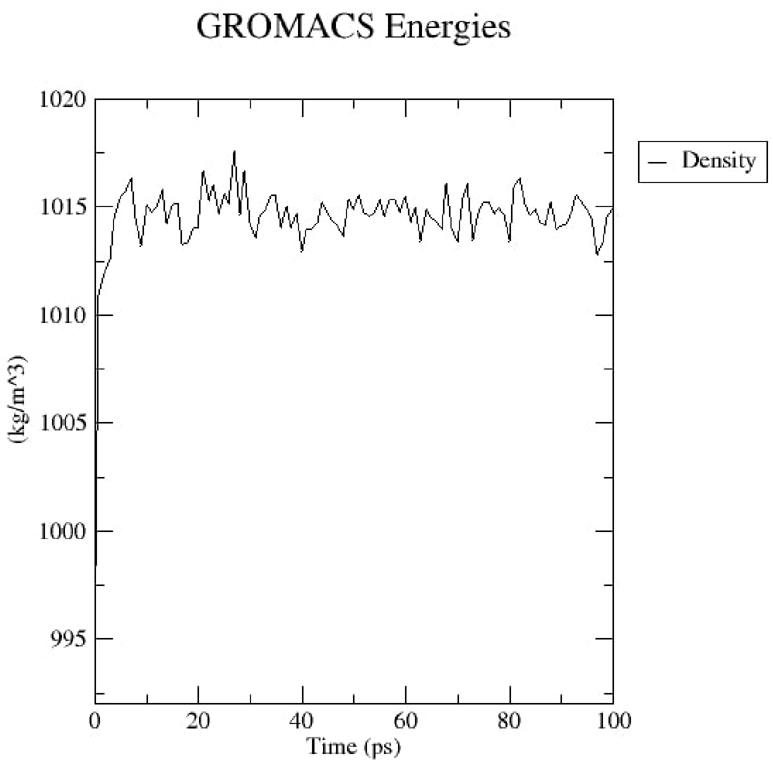

Figure S3. Graph showing minute fluctuations in the system's density during NPT equilibration steps.

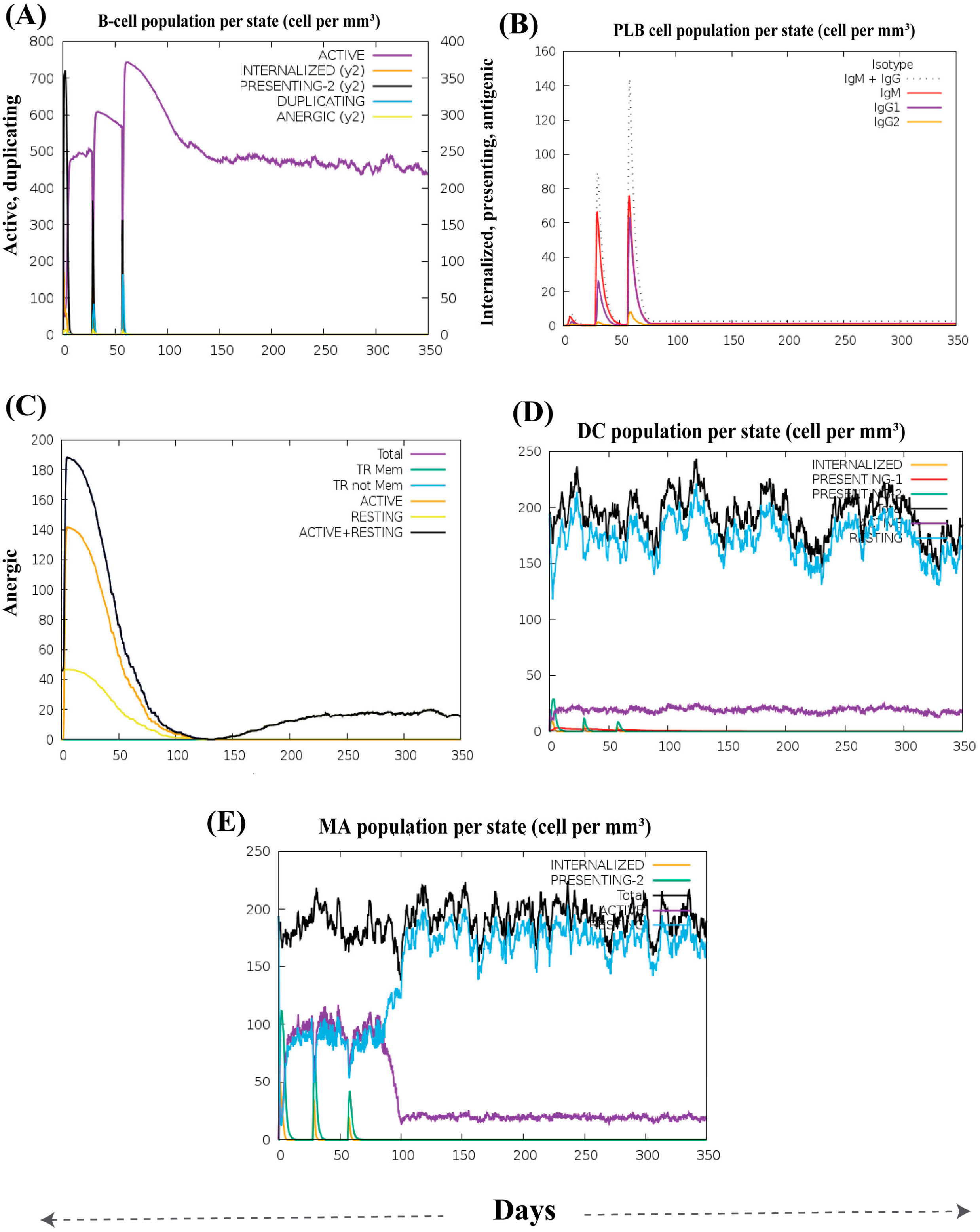

Figure S4. The immunostimulatory potential of the constructed vaccine. **(A)** population/state of active B-cell **(B)** population/state of plasma B-cell and their isotypes **(C)** decrease in the population of regulatory T-cell **(D)** population/state of dendritic cell **(E)** macrophage (MA) activity. All units are given in cells per mm³ in three consecutive immunological responses.

Table S1. Table showing HADDOCK score of top epitope-protein docked cluster along with standard deviation.

HLA class	Finalized epitopes	HLA alleles	RSV protein	HADDOCK score	Standard Deviation (SD)
HLA class I epitopes	NIDIFNPKY	HLA-A*0101	Fusion	-97.1	5.6
	LIAVGLLLY			-90.3	3.6
	IMITTIIIV	HLA-A*0201	Fusion	-85.4	2
	LLHNVNAGK	HLA-A*0301	Fusion	-104.2	3.9
	KTKNTTTTK		Glycoprotein	-113.8	2.4
	ITIELSNIK	HLA-A*1101	Fusion	-105.4	1.7
	KQLLPIVNK			-107.7	3.7
	IASGIAVSK			-105	5.3
HLA class II epitopes	LGFLLGVGSAIASGI	DRB1*0101	Fusion	-93.2	3.3
	LLLYCKARSTPVTLS			-88.9	2.5
	AIIFIASANHKVTLT		Glycoprotein	-117.4	3.3
	AIIFIASANHKVTLT	DRB1*0401	Glycoprotein	-117.1	1.2

Table S2. Table showing the main HADDOCK results of multi-epitope with TLR4. It includes the docking score and all other properties along with their standard deviations.

Parameters	Vaccine-TLR4 cluster
HADDOCK score	-143.3 +/- 9.6
Size of Cluster	20
RMSD from the overall lowest-energy structure	0.3 +/- 0.2
Van der Waals energy	-89.5 +/- 3.9
Electrostatic energy	-371.2 +/- 4.1
Desolvation energy	20.4 +/- 10.0
Restraints violation energy	0.0 +/- 0.00
Total Buried Surface Area	2496.4 +/- 32.1
Z-Score	0

Supplementary File S1: The amino acid sequence of the RSV multi-epitope vaccine (in FASTA format). The adjuvant sequence has been italicized; underlined letters have been used to indicate T-cell epitopes whereas bold letters have been used to indicate the linkers (EAAAK and GPGPG).

>RSV_Vaccine
MTPQNITDLCAEYHNTQIHTLNDKIFSYTESLAGKREMAIITFKNGATFQVEVPGSQHID
*SQKKAIERMKDTLRIAYLTEAKVEKLCVWNNKTPHAIAAISMAN***EAAAK**AIIFIASANHK
VTLT**GPGPG**KTKNTTTTK**GPGPG**ITIELSNIK**GPGPG**LGFLLGVGSAIASGIAVSK**GPGP**
GKQLLPIVNK**GPGPG**NIDIFNPKY**GPGPG**LLHNVNAGK**GPGPG**IMITTIIIV**GPGPG**LIA
VGLLLYCKARSTPVTLS

Supplementary File S2: The optimized codon sequence of the RSV vaccine (in FASTA format).

```
>Optimized_codon_sequence_RSV_vaccine
ATGACCCCGCAGAACATCACCGACCTGTGCGCTGAATACCACAACACCCAGATCCACACC
CTGAACGACAAAATCTTCTCTTACACCGAATCTCTGGCTGGTAAACGTGAAATGGCTATC
ATCACCTTCAAAAACGGTGCTACCTTCCAGGTTGAAGTTCCGGGTTCTCAGCACATCGAC
TCTCAGAAAAAAGCTATCGAACGTATGAAAGACACCCTGCGTATCGCTTACCTGACCGAA
GCTAAAGTTGAAAAACTGTGCGTTTGGAACAACAAAACCCCGCACGCTATCGCTGCTATC
TCTATGGCTAACGAAGCTGCTGCTAAAGCTATCATCTTCATCGCTTCTGCTAACCACAAA
GTTACCCTGACCGGTCCGGGTCCGGGTAAAACCAAAAACACCACCACCACCAAAGGTCCG
GGTCCGGGTATCACCATCGAACTGTCTAACATCAAAGGTCCGGGTCCAGGGCTGGGCTTC
CTGCTGGGTGTTGGTTCTGCTATCGCTTCTGGTATCGCTGTTTCTAAAGGTCCGGGTCCG
GGTAAACAGCTGCTGCCGATCGTTAACAAAGGTCCGGGTCCGGGTAACATCGACATCTTC
AACCCGAAATACGGTCCGGGTCCGGGTCTGCTGCACAACGTTAACGCTGGTAAAGGTCCG
GGTCCGGGTATCATGATCACCACCATCATCATCGTTGGTCCGGGTCCGGGTCTGATCGCT
GTTGGTCTGCTGCTGTACTGCAAAGCTCGTTCTACCCCGGTTACCCTGTCT
```
